# Reply to the letter to “Cultural adaptation and reliability assessment of the Hammersmith neonatal neurological examination for Brazilian newborns at risk of cerebral palsy”

**DOI:** 10.1055/s-0045-1802323

**Published:** 2025-02-06

**Authors:** Mayara Thais Correr, Luzia Iara Pfeifer

**Affiliations:** 1 Universidade de São Paulo, Faculdade de Medicina de Ribeirão Preto, Departamento de Neurociências e Ciências do Comportamento, Programa de Pós-Graduação em Neurologia, Ribeirão Preto SP, Brazil

Dear Editors,

Please find the answers to the questions below.

1. We would like to inform you that the second form of the HNNE published was the incorrect one, and that an erratum needs to be published at least with a reference to the correct one (reference no. 6). 1 Please note that Mac Keith was wrongly spelt in the references list.

**Answer:** We sent a letter to the Journal explaining the alterations and inserted the modifications in the paper. Both are attached.

2. In theory you need permission to publish in a paper, any copy of the HNNE (or HINE) proforma. However, as you do not change it, but only truncate it, this is not a big issue. It would be helpful to add a reference in the Figure legend to our website “ https://www.mackeith.co.uk/hammersmith-neurologicalexamina 'ons/” where the latest versions of the proformas are freely available. However, we no longer use this old version and recommend that published by Romeo in 2012 2 –see next point.

**Answer:** We have included this information in the paper. We attached the paper with the alterations and sent it to the journal.

3. We also still wonder why you used this very old version of the short HNNE from 1999. We understand that this is what you sent to Profs. Dubowitz and Mercuri but they were just giving you permission to translate the proforma that you sent them. The definitive version of the short (screening) HNNE which compared data to the full HNNE data was published by Romeo (2012). 2 As your paper is published 12 years later than this you would have had no problem in using your translation for the items in the Romeo version which would have been more useful. However, as you only use this “old version” for translation purposes and to test intra- and inter-reliability it is not a main issue, but it would be good to clarify in your paper that for clinical purposes the optimal version to use is Romeo (2012). 2

**Answer:** We have included this information in the paper. We attached the paper with the alterations and sent it to the journal.

4. Please also correct the information on page 2 of your paper about which version of the shorter HNNE contains 25 items. It is not the Mercuri version from 2005 that you refer to. The older version you use is only available to people who buy the book from 1999, which is not so easy to do and quite expensive, whereas the other two versions are available as published papers and are far more accessible.

**Answer:** We have included this information in the paper. We attached the paper with the alterations and sent it to the journal.

5. We still find that the use of the term “expanded”, referring to the original full HNNE, is confusing as that is not a term used by the authors of the HNNE. Your paper is published in English, and the readership will not be only from “your culture”. Anyone looking up this term will not find it anywhere else to be associated with the HNNE and will wonder if you are referring to another version of the HNNE.

**Answer:** We have included this information in the paper. We attached the paper with the alterations and sent it to the journal.

6. At the end of the first paragraph on page 3 you say “both versions can be applied to premature infants” but when using the findings in the first and last columns as abnormal, preterm infants need to have reached term-equivalent age.

**Answer:** We include in the paper the information “respecting corrected age for application in preterm babies”.

7. We have no issues with your translation process. However, you do not say whether your translation is materially different from that already available on the Mac Keith website. The change of title seems fine.

**Answer:** According to the process you have followed, our translation and cross-adaptation were carried out with all the scientific rigor necessary for the instrument to be used in our population. There was no free translation. In addition, the translation published on the Mac Keith website was published after your team authorized our research and was only made available on the site when we were finalizing the whole process. Your team already had access to our translation when this happened since the translation was the first stage of development.

8. We stress that although the HNNE can be translated, it does not require cultural adaptation or psychometric analysis. The text on the form must not be altered, using the word “adaptation” sounds as if the text is subtly different even if this is not the case.

**Answer:** Most assessment instruments were developed in countries of the global North and have been misused in countries of the global South due to a lack of cultural equivalence in sociocultural realities that differ from their origin. This factor interferes with the designation of results in different ethnocultural realities. 3 It is well documented in scientific studies that the different clinical manifestations, evolutions, and prognoses are interdependent on the culture in which the subjects are inserted. It is, therefore, essential to standardize an instrument to establish a common language among professionals and researchers and to draw up a worldwide overview of ethnocultural variations and sociocultural factors to the detriment of different regions. 3 Here in Brazil, we have organized the process of cross-cultural adaptation to follow scientific criteria. To this end, before starting our research, we asked the authors of the HNNE and HINE for authorization for this process, and we received this authorization before starting all the stages, as proof sent by email to the journal.

9. The HNNE is a neurological exam and not a psychological test, and as such does not need psychometric analysis.

**Answer:** In this case, the psychometric analysis was carried out to confirm that the cross-culturally adapted version maintained the same reliability as the original version, i.e., the reliability of measuring precisely, consistently, and stably what is proposed and with a high degree of agreement between multiple measures of the same object. Psychometric validation consists of evaluating the reliability and validity measures of the instrument. Reliability is obtained by internal consistency (measured by Cronbach's alpha coefficient) and reproducibility (measured by test and retest). 4 5

We would like to inform you that this research is part of a larger research project called “Translation, cross-cultural adaptation, and validation of the Hammersmith Infant Neurological Assessment (HINE) instrument for Brazilian infants at risk of cerebral palsy” ( *Tradução, adaptação transcultural e validação do instrumento Hammersmith Infant Neurological Assessment (HINE) para lactentes brasileiros com risco de paralisia cerebral 6* ), developed in the PhD program of the Postgraduate Program in Neurology of the Faculdade de Medicina de Ribeirão Preto Universidade de São Paulo. We received the authorization of Dr. Eugenio Mercuri to proceed with this cross-adaptation, and for this research's development, the ethics committee analyzed and approved the project under opinion no. 1.809.858. This thesis was analyzed and approved by three doctoral researchers, and the manuscript was analyzed by two blind reviewers and was approved.
